# LioNeo project: a randomised double-blind clinical trial for nutrition of very-low-birth-weight infants

**DOI:** 10.1017/S0007114521005110

**Published:** 2022-12-28

**Authors:** Vicky Nogueira-Pileggi, Maria Carolina Achcar, Fábio Carmona, Adriana Carnevale da Silva, Davi Casale Aragon, Fabio da Veiga Ued, Mariana Moraes de Oliveira, Luciana Mara Monti Fonseca, Larissa Garcia Alves, Vanessa Silva Bomfim, Tania Maria Beltramini Trevilato, Mayara Condé Brondi Delácio, Cyntia Takeko Amorim Minakawa de Freitas, Viviane dos Santos Porto, Daniela de Castro Barbosa Leonello, Natalia de Paiva Martins, Heloisa Gasparini Marigheti Brassaro, Marisa Márcia Muyssi-Pinhata, José Simon Camelo Junior

**Affiliations:** 1 Department of Pediatrics, Ribeirão Preto Medical School, University of São Paulo, São Paulo, Brazil; 2 Department of Maternal-Infantile and Public Health, Nursing School of Ribeirão Preto, University of São Paulo, São Paulo, Brazil; 3 Human Milk Bank, Clinics Hospital, Ribeirão Preto Medical School, University of São Paulo, São Paulo, Brazil; 4 Laboratory of Pediatrics, Division of Metals and Rare Diseases, Clinics Hospital, Ribeirão Preto Medical School, University of São Paulo, São Paulo, Brazil; 5 Children’s Hospital, Clinics Hospital, Ribeirão Preto Medical School, University of São Paulo, São Paulo, Brazil

**Keywords:** Human milk, Human milk bank, Preterm newborn, Lyophilisate, Very-low-birth-weight infants, Necrotising enterocolitis

## Abstract

We assessed the effectiveness of lyophilised banked human milk (HM) as a fortifier to feed very-low-birth-weight infants (VLBWI). This study aimed to evaluate the safety and tolerability of HM with HM lyophilisate as an additive compared with the standard additive (cows’ milk protein). In this phase I double-blind randomised controlled clinical trial, set in the intensive and intermediate care units of a tertiary hospital, forty VLBWI were enrolled and allocated into two groups: HM plus HM lyophilisate (LioNeo) or HM plus commercial additive (HMCA). The inclusion criteria were preterm infants, birth weight 750–1500 g, small or adequate for gestational age, exclusively receiving donor HM, volume ≥ 100 ml/kg per d and haemodynamically stable. Participants were followed up for 21 consecutive days. The primary outcome measures were necrotising enterocolitis (NEC), late-onset sepsis (LOS), death, gastrointestinal (GI) bleeding or perforation, diarrhoea, regurgitation, vomiting and abdominal distension. The LioNeo and HMCA groups had similar weights at baseline. The regression models showed no differences between the groups in terms of the primary outcomes. Diarrhoea, GI perforation, NEC and LOS were absent in the LioNeo group (one LOS and one NEC in the HMCA group). Multiple regression analysis with the total volume of milk as a covariate did not show significant differences. The lyophilisation of donor HM was considered safe and tolerable for use in stable haemodynamically VLBWI.

Breast-feeding and human milk (HM) are the best options for newborns and preterm newborns^(1–3)^. An optimal nutritional approach is a key to increasing the survival rate of newborns^(4,5)^. However, very-low-birth-weight infants (VLBWI) may not receive an adequate volume of HM to meet their nutritional demands. Such nutritional deficits can be overcome using HM additives or fortifiers made from hydrolysates of cows’ milk protein. Such additives promote the growth of VLBWI, fortifying some essential nutrients in raw or banked HM (such as protein, Na, Ca and P), thereby significantly improving their nutritional value. Despite the potential benefits, commercial additives may alter the immunological properties of HM, similar to the Holder pasteurisation process, and increase the risk of sensitisation by heterologous protein, increasing the risk of gastrointestinal (GI) bleeding, necrotising enterocolitis (NEC) and late-onset sepsis (LOS).

Several studies have examined HM fortifiers that can provide excellent nutrition to VLBWI. HM can provide non-nutrient immunological components that can contribute to the integrity of the GI mucosa and increase immunity against several different infections^(6–8)^. Our group recently developed a lyophilisate from donated HM exclusively from HM banks to be used as an additive, with safe component characteristics that can be used in VLBWI^(9–11)^.

We, therefore, hypothesised that lyophilisation of HM is safe and tolerable in the nutrition of VLBWI. This study aimed to assess the safety and tolerability of HM with HM lyophilisate as an additive compared with the standard additive (based on hydrolysed cows’ milk protein).

## Methods

### Study design and setting

This was a double-blind, randomised controlled clinical trial approved by the Ethics Committee of the Clinics Hospital of Ribeirão Preto Medical School (Brazil) CAAE: 96682318.2.0000.5440, and written informed consent was obtained from all participants’ parents or guardians. We tested the safety and tolerability of a control group using a gold standard for VLBWI. The trial was registered at the Universal Trial Number (UTN): U1111-1220-0550 – http://www.ensaiosclinicos.gov.br/rg/RBR-8nnpfm/. The study was performed in a tertiary hospital in neonatal intensive and intermediate care units.

We decided to use a control group for phase 1 because it would be unethical to use it in normal newborn infants who were breastfed and did not require a fortifier. Therefore, our population of choice was VLBWI in their most stable state. This population usually uses a fortifier that is accepted worldwide.

### Patient eligibility

The inclusion criteria were preterm birth (< 37 weeks gestational age), birth weight ≥ 750 g and ≤ 1500 g, small or adequate for gestational age receiving exclusively HM at a volume of ≥ 100 ml/kg per d, haemodynamically stable (no vasoactive drugs, blood pressure within the normal range and/or good peripheral perfusion, a capillary filling time less than 3 s, palpable peripheral pulses and oxygen saturation greater than 90 %) and whose parents or legal guardians signed the informed consent. It was important to clarify that most neonates receive donated HM at this stage and some of them use donated plus HM from their own mothers. In the case of identical twins, only one of the newborns could be enrolled in the study. Both the fraternal twins were eligible. The exclusion criteria included large for gestational age (because they tended to grow faster because of their potential hyperinsulinism), major malformations (congenital heart diseases) and grade III and IV peri- or intraventricular haemorrhages.

### Intervention and control groups

In summary, the following steps were performed in the preclinical phase: 50 ml of HM was lyophilised to yield 7 g of powdered HM, which was then reconstituted in 75 ml of HM from the donor, both raw, creating a concentrate of HM called LioNeo (online Supplement 1). The energy content ranged from 119,5 to 167,3 kJ/l (500 to 700 kcal/l), and the Dornic acidity was < 8°D (within normal range). The whole HM was successfully freeze-dried without any preparatory steps. This concentrate did not exceed the osmolality of 450 mOsm/kg H_2_O and was subjected to pasteurisation and microbiological quality control. After adding the lyophilisate (immediate concentrate) to HM, the resulting LioNeo was tested for its properties. The tests were repeated after storage of the concentrate in a freezer for 3 and 6 months^(7,8)^. Macronutrients were quantified using IR spectroscopy, osmolality and lipid profile by GC, and micronutrients were quantified using atomic absorption spectrophotometry at four different moments (HM baseline, HM concentrated and HM concentrated stored during 3 and 6 months). We also tested for and ruled out the presence of heavy and potentially toxic micronutrients using inductively coupled plasma-MS^(9)^. All preclinical tests proved that this new additive could be tested in VLBWI.

The intervention consisted of feeding the infants randomised to group A with LioNeo (75 ml of HM plus 7 g of powdered HM). Control group B was fed with HM plus an additive from cows’ milk protein origin (FM 85^®^, Nestlé^®^) at a concentration of 4 % HM plus commercial additive (HMCA). Both groups were followed up in a similar manner.

Three unblinded nutritionists were responsible for preparing the nutrition according to the daily prescription. Diet for both groups had the same appearance and smell and could not be distinguished at the bedside. The main differences between the additive components are listed in Table 1. Both preparations were formulated in the hospital’s milk room, respecting all safety and best practices for HM manipulation. The milk samples were portioned into syringes and fed for 24 h. The nutritionists responsible for portioning the milk were also tasked with transport and storage in a separate refrigerator. The neonatal unit nurses were responsible for administering the milk from the syringe into the infusion pump.

**Table 1.** Energy, macro and micronutrients per 100 ml of human milk baseline (HM-baseline), human milk concentrated with lyophilisate of human origin (LioNeo – intervention group) and human milk enriched with commercial additives based on hydrolysed cows’ milk protein (FM 85^®^, Nestlé^®^ – control group) at a concentration of 4 %* † (Mean values and standard deviations)

Components of human milk with and without additives						
			Intervention group		Control group	
	HM-baseline		LioNeo		HM baseline + FM85 4 % (HMCA)	
Energy and nutrients	Mean	sd	Mean	sd	Mean	sd
Energy (kcal)	56·3	10·5	80·0	13·7	73·5	10·5
Protein (g)	0·9	0·5	1·5	0·6	2·3	0·5
Carbohydrate (g)	7·1	0·7	9·2	0·7	8·4	0·7
Total lipids (g)	2·6	1·1	4·0	1·4	3·3	1·1
Ca (mg)	23·2	4·7	36·5	7·2	99·2	4·7
Mg (mg)	2·3	0·5	3·7	0·7	6·3	0·5
Na (mg)	13·1		22·3	16·9	49·9	9·1
K (mg)	60·1	14·8	101·4	26·9	108·1	14·8
Cu (µg)	33·4	14·6	48·3	19·9	85·4	14·6
Zn (mg)	0·1	0·1	0·2	0·1	1·1	0·1
P (mg)	14·8	6·0	18·5	6·0	58·5	6·3
Fe (mg)	0·1	0·0	0·1	0·0	1·9	0·0

* We used as standard mature human milk (HM – baseline) to prepare the additives to the intervention group.

† It is important to stress that all micronutrients were monitored by the medical staff and as needed.

All neonates started to receive the project’s diet after the enteral feeding reached a volume of at least 100 ml/kg of body weight and were followed up for 21 consecutive days (maximum volume 160 ml/kg per d)^(10,11)^. The medical staff decided on any feeding interruption based on the nutrition protocol of neonatal unity. The project team did not interfere with medical decisions (the feeding protocol used in the hospital can be found in online Supplementary File 2).

Before beginning the study, the enteral nutrition recommendations for preterm newborns were reviewed with the clinical staff to update the norms to start feeding, progression and management of abdominal occurrences. The infants were cared for by medical staff and nurses, and the routine management guidelines did not change. The medical staff decided on the volume, change or the interruption of the diet, if necessary.

### Randomisation and masking

All participants were randomised by a computer-generated list into one of the groups by simple randomisation at a ratio of 1:1. Allocation concealment was granted by Research Electronic Data Capture (REDCap). The minimum sample size required by the NIH/FDA of twenty patients^(12)^ for each group in phase 1 studies. The participants’ parents or guardians, care providers (nurses, doctors and hospital staff), investigators and outcome assessors were blinded to study criteria. Only the three nutritionists responsible for portioning the milk in the syringes were unblinded.

### Outcomes measurements

The patients were checked daily by a research nurse to perform the measurements and to fill up the form in the REDCap database and electronic hospital medical records. REDCap is a metadata-driven application that aims to support translational research projects in academic environments. Initially developed by Vanderbilt University, REDCap is currently supported by 398 partner institutions in forty-six countries (Vanderbilt University, 2012) and supports nearly 40 000 projects worldwide. More information can be obtained from the following website: https://www.project-redcap.org/.

The daily assessment consisted of measuring body weight, abdominal circumference, the volume of actually ingested milk and the main outcome events. All data were then double-checked by the project manager, and any inconsistencies were resolved by the end of the week. If the participant had any adverse event, a physician of the research team (blinded) would monitor it closely along with the medical staff.

The main outcomes and definitions were:

- Diarrhoea: increase in the liquid content of the stool or number of evacuations outside the normal pattern observed in the neonate.
- Vomit: an abrupt return of a large volume of gastric content (milk) to the mouth.
- Regurgitation: an abrupt return of a small volume of gastric content (milk) to the mouth.
- Abdominal distension: any increase in abdominal circumference of ≥ 10 %, independent of clinical repercussions or significance.
- GI bleeding: melena (dark blood in the stools, reminding coffee grounds), enterorrhagia (red living blood in the stools) and haematemesis (vomiting blood).
- GI perforation: clinical and/or radiological signs of GI perforation, with free air into the peritoneal cavity, without any sign of NEC, which should be ruled out.
- NEC: Bell Grade 1 B or more severe criteria verified by clinical method, serial radiographs of the abdomen, ultrasound of the abdomen, blood counts, PCR and blood cultures. Eventually, an exploratory laparotomy was performed. The average incidence described in the literature ranges from 3·2 % to 10·9 %^(13,14)^, but notwithstanding, any NEC event was considered a serious adverse effect.
- Sepsis: the following symptoms can be seen: general symptoms (fever, thermal instability, oedema, malnutrition, poor appearance), GI symptoms (bloating, vomiting, diarrhoea or hepatomegaly), respiratory symptoms (apnoea, dyspnoea, tachypnoea, intercostal retractions, nasal flapping, groaning and cyanosis), renal symptoms (oliguria), cardiovascular symptoms (pallor, mottled, cold or sticky skin, tachycardia, hypotension or bradycardia), central nervous system-related symptoms (irritability, lethargy, tremors, seizures, hyporeflexia, hypotonia, abnormal Moro reflex, irregular breathing, bulging fontanelle and abnormal crying), haematological signs (jaundice, splenomegaly, pallor, petechiae, purpura and bleeding), laboratories examination results (blood count with leukocytosis, left shift or leukopenia, with qualitative changes such as toxic granulation). Identification of bacteria or fungi by blood, cerebral spinal fluid or urine cultures were also included.
- LOS was confirmed by compatible culture, and neonates were divided into suspected sepsis and confirmed according to this criterion: action: suspension of diet until complete haemodynamic stability returns, if associated with NEC, for at least 7 days + antibiotic therapy.

We also compared the days of feeding intake between the two groups. It is important to note that the intake of mother’s milk (MOM) was prioritised and stimulated.

### Safety and tolerance

We tested safety as the appearance of NEC, death, sepsis, septic shock or GI bleeding. Tolerance was defined as vomiting, diarrhoea, abdominal distension or suspension of diet for any period.

### Statistical analysis

A detailed exploratory data analysis is reported in the tables containing measures of central tendency, dispersion, and absolute and relative frequencies. Simple and multiple log-binomial regression models were fitted to estimate the relative risks of adverse events between the groups and 95 % CI. Multiple models were adjusted for the total volume of ingested milk as a covariate throughout the 21 d.

When comparing the averages of the primary outcomes (vomit, regurgitation, abdominal distension, GI bleeding, GI perforation, NEC and sepsis), accumulated by an individual (considering total events counted during 21 d), between the groups, we performed simple and multiple regression analyses based on the double Poisson distribution. The multiple regression model was fitted by considering the volume of ingested milk as a covariate. This model is more suitable for counted data because it does not assume that the mean must be equal to the variance, as in the standard Poisson model. Daily milk intake was compared using a linear regression model. The software used was SAS 9.4 (SAS Institute).

### Interim analysis

During the study, events occurring in the trial were systematically reviewed by the study investigators in a blinded manner and classified according to the WHO guidelines^(15)^.

Interim analysis was performed on twenty patients. An independent data safety monitoring board, composed before the trial began, was responsible for analysing unblinded data and deciding whether the trial should continue. The frequencies of adverse events for possible interruption of the study were defined according to the Vermont Oxford Network^(16)^. All three members agreed that the trial should be continued.

## Results

**Fig. 1.**
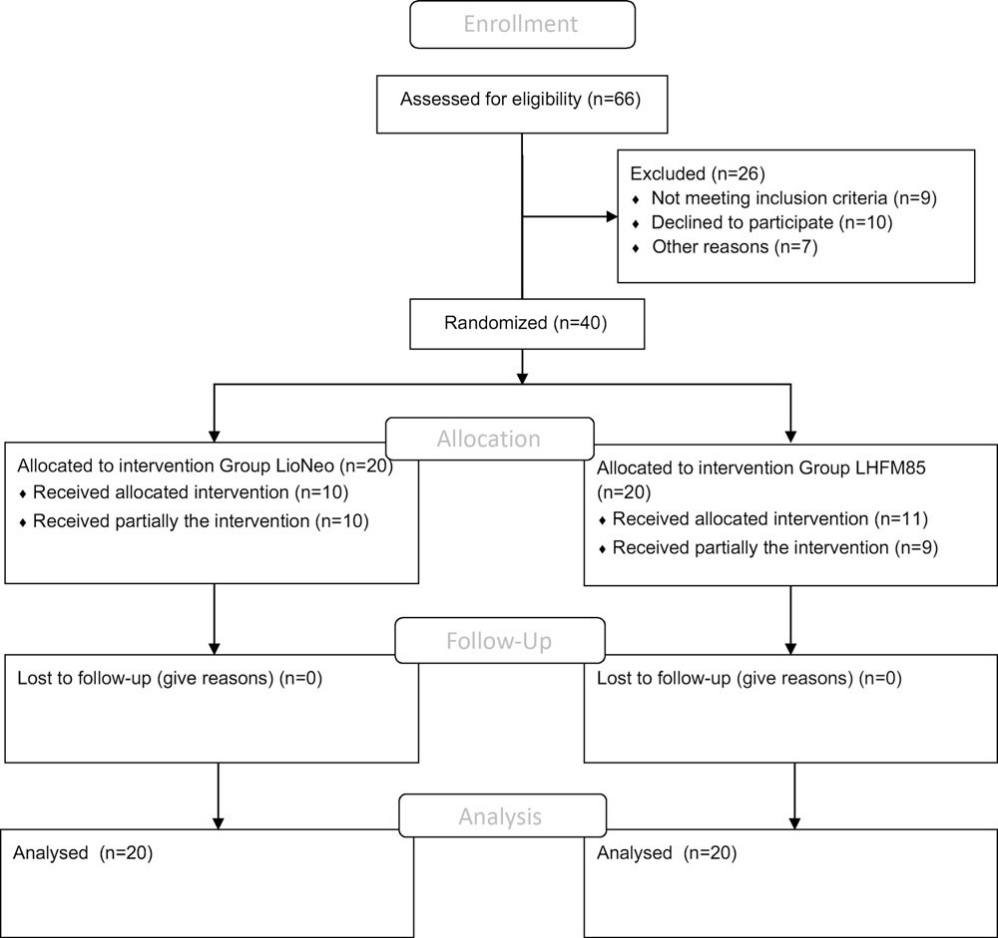
Flow chart of included neonates.

Sixty-six patients were screened, and forty neonates were included from 6 May 2019 to 6 April 2020. Data from all participants were collected and entered into REDCap, according to the flow chart shown in Fig. 1. These forty infants were randomly assigned to two groups (intervention group = LioNeo, *n* 20, and control group = HMCA, *n* 20). Their main characteristics are presented in Table 2. There were eleven girls in LioNeo (52·38 %) and 10 (50 %) girls in the HMCA group.

**Table 2.** Characterisation of the preterm infants according to the group. LioNeo (intervention group); HMCA (control group) (Mean values and standard deviations)

	Intervention group					Control group				
	Mean	sd	Q1	Median	Q3	Mean	sd	Q1	Median	Q3
Birth weight (g)	1220·00	200·69	1090·00	1242·50	1393·00	1219·30	204·78	1063·00	1255·00	1373·00
Gestational age (weeks)	30·45	2·68	28·50	30·50	32·50	29·70	1·75	28·50	30·00	31·00
Birth lenght (cm)	38·08	2·17	36·00	38·00	40·00	37·77	2·89	36·00	39·00	40·00
Birth head circumference (cm)	27·50	1·67	26·25	28·00	29·00	27·03	1·41	25·50	27·50	28·50
Age at inclusion (days	11·40	8·92	6·00	9·50	12·00	12·05	6·44	7·00	10·00	16·50
Weight (day 1)	1222·80	178·09	1105·00	1210·00	1355·00	1254·30	200·43	1185·00	1275·00	1388·00
Length (day 1)	38·46	2·33	36·90	38·50	39·85	38·85	2·20	37·30	38·75	40·25
Head circumference (day 1)	26·34	2·13	24·60	26·00	28·40	26·27	1·99	25·30	26·45	27·75
Weight (day 21)	1565·30	263·79	1400·00	1557·50	1728·00	1678·50	262·60	1510·00	1687·50	1890·00
Length (day 21)	41·14	1·92	39·80	41·25	43·00	42·05	2·14	40·80	42·25	43·35
Head circumference (day 21)	28·54	2·29	26·30	29·10	30·10	28·39	2·07	27·00	28·65	30·20

Regarding intervention, twenty patients received total prescribed volume in 21 days, the average for LioNeo was 18 d (median = 21 d) and HMCA was 17·35 d (median = 20·5 d), with no significant difference.

Among the twenty infants in group B, the following occurrences were registered: diarrhoea (1), GI perforation (1), NEC (1) and clinical LOS (1). These outcomes were not analysed or statistically compared between the groups because these events did not occur in group A. Other outcomes were analysed and are presented in Tables 3 and 4.

**Table 3.** Results of adjustments of the regression models, single and multiple, based on the double Poisson distribution, comparing averages (intervention group *minus* control group) of adverse event counts, by individual (Mean values and 95 % confidence intervals)

	Simple model		Multiple model	
Variable	Difference between means	95 % CI	Difference between means	95 % CI
Regurgitation	0	–0·13, 0·13	0	–0·06, 0·06
Vomit	–1·00	–2·25, 0·25	–2·02	–5·05, 1·00
Abdominal distension	–0·35	–1·23, 0·52	–0·45	–1·75, 0·84
Gastrointestinal bleeding	0·10	–0·56, 0·76	1·41	–3·03, 5·85
Number of days receiving diet	0·65	–2·65, 3·95		
Volume of diet received (ml)	191·80	–603·72, 987·31		

**Table 4.** Incidence of outcomes of interest at the end of the study, according to the group and results of adjustments to the log-binomial regression models, simple and multiple. The results are expressed in absolute numbers of adverse events. LioNeo (intervention group); HMCA (control group) (relative risks and 95 % confidence intervals; numbers and percentages)

	Group								
	Intervention (*n* 20)			Control (*n* 20)					
Outcome	*n*	%		*n*	%	RR	95 % CI	RRaj	95 % CI
Diarrhoea	0		0·00	1	5·00	*		*	
Regurgitation	2		10·00	2	10·00	1·00	0·16, 6·41	0·95	0·14, 6·08
Vomit	12		60·00	10	50·00	0·83	0·47, 1·47	0·93	0·51, 1·69
Abdominal distension	16		80·00	16	80·00	1·00	0·73, 1·37	0·99	0·70, 1·39
Gastrointestinal bleeding	2		10·00	5	25·00	2·50	0·55, 11·40	4·94	0·69, 35·12
Gastrointestinal perforation	0		0·00	1	5·00	*		*	
Necrotising enterocolitis	0		0·00	1	5·00	*		*	
Neonatal sepsis	0		0·00	1	5·00	*		*	

RR, relative risk; RRaj, relative risk adjusted for the total volume of milk ingested throughout the group.

Incidence: if the individual experienced an event at least once, it was classified as positive for that event.

* It was not possible to estimate the relative risk due to the presence of sampling zeros.

Table 3 shows no differences between means in the events that appeared in either group. Table 4 describes all outcomes analysed, showing no difference in those that appeared in both groups. It is worth highlighting that some events only appeared in the HMCA group.

The comparison of the duration of milk ingestion between groups had a mean difference of 0·65 (95 % CI −2·65, 3·95) and a *P*-value of 0·70. We also compared the means of the total volume ingested, and no difference was found (191·80; 95 % CI −603·72, 987; *P* = 0·63).

**Fig. 2.**
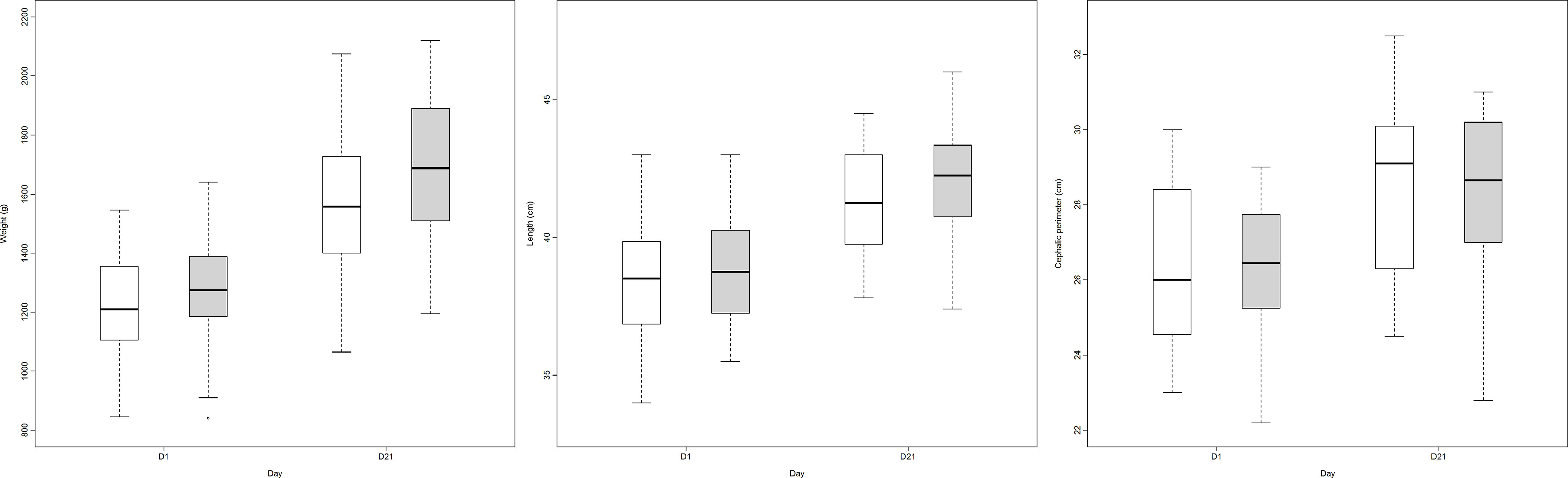
Growth (weight, length and head circumference) in both groups comparing day 1 and day 21 (group A: LioNeo; group B: HMCA). 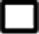, intervention group; 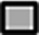, control group.

Finally, Table 5 describes the variables studied for 21 d. A boxplot of the anthropometric data during the study is shown in Fig. 2.

**Table 5.** Description of the variables of interest during the follow-up according to the group. LioNeo (intervention group); HMCA (control group) (Mean values and standard deviation; median values and quartiles)

	Intervention group					Control group				
	Mean	sd	Q1	Median	Q3	Mean	sd	Q1	Median	Q3
Weight variation (g)	342·50	115·62	295·00	327·50	427·50	424·25	125·71	352·50	437·50	507·50
Length variation (cm)	2·68	1·32	1·50	2·65	3·75	3·46	1·30	2·50	3·30	4·20
Head circumference variation (cm)	2·74	1·69	1·50	2·20	3·20	2·25	1·40	1·40	2·20	3·00
Volume of ingested milk (ml)	3415·20	1426·50	2432·00	3947·00	4437·00	3607·00	1026·50	2930·00	3679·00	4466·00
Days of milk intake	17·35	5·98	16·00	20·50	21·00	18·00	4·17	14·00	21·00	21·00
Average milk intake per day	192·05	35·95	171·70	200·95	212·10	201·02	39·16	167·30	212·64	225·60

Variation is from day 1 to day 21.

Other analyses that were not part of the initial protocol, such as frequency of feeding, comparison between groups and intragroup of weight, height and head circumference and velocity of daily weight gain, length and head circumference, which were not part of the objectives of phase 1, can be found in online Supplementary File 3.

All data are available in the hospital electronic documents, and they can be audited as needed.

## Discussion

To the best of our knowledge, this is the first study to compare concentrated banked HM using a lyophilisate from HM as an additive in preterm VLBWI for safety and tolerability. The study found no statistical differences in the incidence of adverse outcomes between the groups. Therefore, the banked HM fortifier was considered safe and tolerable for use in this population. It is noteworthy that no infants in the LioNeo group suffered diarrhoea, GI perforation, NEC or LOS.

HM is the preferred nutrition for preterm VLBWI. There is strong support encouraging mothers to breast-feed. However, this can often be a challenge during a hospital stay, considering the immature characteristics of VLBWI. Donor HM is a safe alternative when the MOM is not available^(17,18)^. Thus, to provide the best nutrition to the infant, it is common to use banked HM along with the mother’s raw milk^(19)^. There have been some recent attempts to concentrate HM up to 30 % of the nutrients, which we consider an advanced approach^(19)^. Our method was able to concentrate on an average of 60 % nutrients^(10)^.

Comparing the LioNeo with the commercial additive, the length, weight and head circumference showed no differences despite the differences in the protein content of the additives. This could lead to a complex discussion about nutrition in VLBWI as a whole, regarding the importance of all nutrients and not only the protein level. All babies were tested during the hospital stay for micronutrients, and any micronutrient deficiencies were supplemented with adequate nutrition as needed. It is important to note that some babies did not receive the 21-d straight intervention due to abdominal distension or vomiting (probably related to the higher osmolality of both additives) with no clinical relevance.

A recent Japanese guideline assures that the best nutrition for VLBWI is their MOM, and, if not possible, donor HM should be used^(20)^. They also predicted that it would be necessary to supply an exclusive HM-based diet for these infants in the future, including a milk-derived HM fortifier. Using human donor milk is a more cost-effective measure, as shown in the Editorial article by Verduci et *al.* when they compared medical and non-medical costs^(21)^.

This methodology is inexpensive and can be performed on HM banks worldwide. Although there is a human protein additive on the market, the costs are high and involve ethical issues due to the HM trade. The cost was dependent on the weight-specific feeding scheme and was calculated at a price of €6 per ml for the fortifier^(22)^. The use of donor milk from HM banks is a strength of our study because it guarantees the gratuity and non-commercialisation of the product that might benefit preterm babies and prevent serious consequences of undernutrition in this population^(23)^.

To predict safety and tolerability, the primary adverse outcomes associated with the use of fortifiers were closely observed. A recent study (2016) reported that using donor milk for preterm infants leads to a 10 % increase in breast-feeding and a 2·6 % decrease in NEC incidence rates^(24)^. A Cochrane review comparing formula *v*. donor breast milk for feeding preterm or low birth weight infants found a lower incidence of NEC in the donor breast milk (relative risk 1·87, 95 % CI 1·23, 2·85)^(25)^.

Neonatal sepsis is one of the leading causes of neonatal deaths worldwide. According to global trends, this corresponds to 2·6 % of all deaths^(1)^. VLBWI are at an increased risk due to their immature immune system and hospitalisation. A previous study showed that the risk factors were gestational age and maternal exposure to antenatal antibiotics^(26)^. There were no cases of NEC and LOS in the LioNeo group, and because of the incidence in only one group, no comparative analysis could be performed. Nevertheless, this fact could suggest that concentrated HM, using HM lyophilisate, can protect against the adverse outcomes observed in this study.

Recently, Dani et *al.*^(27)^ hypothesised that enteral feeding induces mesenteric haemodynamic changes in preterm infants, which may vary according to the milk used. They evaluated changes in regional splanchnic oxygenation measured by near-IR spectroscopy in infants fed with MOM, fortified human milk (FHM) or preterm formula. They observed that splanchnic oxygenation was not affected by MOM feeding, was transiently decreased by FHM feeding and was persistently decreased by preterm formula. These results suggest that preterm infants who received preterm formula had higher splanchnic tissue oxygen extraction than those who received MOM or FHM. FHM with a product of bovine origin transiently increased the splanchnic energy expenditure. These findings suggest an increased risk of NEC and GI bleeding due to cows’ milk protein exposure, especially from preterm formula.

Both groups were fed for a similar period in our study, with no differences between the means. The fasting in our study was primarily due to GI problems, such as abdominal distension and vomiting. Both are very common in preterm newborns, especially in infants using antibiotics^(28)^. In addition, preterm infants, due to the incomplete development of the GI motility system, are more susceptible to abdominal distention^(29)^. Feeding intolerance, such as regurgitation, vomiting and abdominal distension, could cause weight loss, increase the risk of infection and prolong prenatal feeding^(29)^. It is common practice to stop enteral feeding in VLBWI who do not tolerate it.

Despite its potential, the European Milk Bank Association Working Group reports that it is early to conclude the use of human fortifiers in premature babies^(30)^. However, this could generate valuable insights once phase 2 of this study with a larger sample size is started. In addition to efficacy, adverse outcomes will continue to be observed in the next part of the project, enhancing safety and tolerability.

Our study had some limitations. The small sample size made it impossible to compare some of the primary outcomes that were less prevalent, such as NEC and LOS. We hope to have a better sample in phase 2; however, it is noteworthy that there were no serious adverse outcomes reported in the LioNeo group, and there were some isolates in the control group. Another limitation was the number of days of ingestion in all included newborns. It is worth noting that most of them completed at least 15 days of use. Therefore, we believe that, in reality, feeding has been temporarily stopped in some VLBWI due to dysmotility, secondary to their immaturity.

### Conclusions

This study is notable for being one of the few randomised controlled trials performed among stable preterm VLBWI aiming to improve their nutrition with FHM and keep them safe from adverse outcomes that might occur during their time in hospital. We tested the fortifier based on mature HM, and the results were promising, showing that the main outcomes were not different from the standard fortifier currently used based on cows’ milk. Therefore, we conclude that this new human-based fortifier is safe and tolerable for use in this population, and we are now moving forward to phase 2.

## Supplementary material

For supplementary material accompanying this paper visit https://doi.org/10.1017/S0007114521005110. click here to view supplementary material
