# Supplementary material for: LioNeo project: a randomised double-blind clinical trial for nutrition of very-low-birth-weight infants
Source: Br J Nutr. 2022 Feb 11;128(12):2490–7. doi: 10.1017/S0007114521005110 (PMC9723485; doi:10.1017/S0007114521005110)
Supplement: Supplementary file 1 [file S0007114521005110sup.zip › S0007114521005110sup003.docx]

**Supplement 2**

| Days of Feeding | Control Group (%) | Intervention Group (%) | Total |
| --- | --- | --- | --- |
| 4 | 2 (10) | 0 (0) | 2 |
| 8 | 1 (5) | 0 (0) | 1 |
| 9 | 1 (5) | 1 (5) | 2 |
| 11 | 0 (0) | 1 (5) | 1 |
| 12 | 0 (0) | 1 (5) | 1 |
| 13 | 0 (0) | 2 (10) | 2 |
| 15 | 1 (5) | 1 (5) | 2 |
| 17 | 1 (5) | 1 (5) | 2 |
| 19 | 0 (0) | 1 (5) | 1 |
| 20 | 4 (20) | 1 (5) | 5 |
| 21 | 10 (50) | 11 (55) | 21 |
| TOTAL | 20 | 20 | 40 |

Comparisons between the means of the groups - linear mixed effects model (adjusted for the average daily volume ingested).

| Variable | Comparison | Difference between means | p-value | Lower limit IC95% | Upper limit IC95% |
| --- | --- | --- | --- | --- | --- |
| Weight | AD1 - AD21 | -342,50 | < 0,01 | -397,17 | -287,83 |
|  | BD1 - BD21 | -424,25 | < 0,01 | -478,92 | -369,58 |
|  | AD1 - BD1 | -0,66 | 0,99 | -124,40 | 123,07 |
|  | AD21 - BD21 | -82,41 | 0,19 | -206,15 | 41,32 |
| Height | AD1 - AD21 | -2,68 | < 0,01 | -3,37 | -1,99 |
|  | BD1 - BD21 | -3,20 | < 0,01 | -3,89 | -2,51 |
|  | AD1 - BD1 | -0,18 | 0,78 | -1,46 | 1,11 |
|  | AD21 - BD21 | -0,70 | 0,28 | -1,98 | 0,59 |
| Head circumference | AD1 - AD21 | -2,20 | < 0,01 | -3,01 | -1,38 |
|  | BD1 - BD21 | -2,12 | < 0,01 | -2,93 | -1,30 |
|  | AD1 - BD1 | 0,31 | 0,61 | -0,91 | 1,53 |
|  | AD21 - BD21 | 0,39 | 0,52 | -0,83 | 1,61 |

A: Grup A = Control Group; B: Intervention Group; D1: day 1; D21: day 21.

Velocity of Weight, Length, Head Circumference between groups using Mann Whitney non-parametric test

| Group | n | Velocity | Mean | Standard Deviation | Q1 | Median | Q3 |
| --- | --- | --- | --- | --- | --- | --- | --- |
| Intervention | 20 | Weight | 19.38 | 19.89 | 10.81 | 13.55 | 16.15 |
|  |  | Length | 0.89 | 0.44 | 0.50 | 0.88 | 1.25 |
|  |  | Head Circumference | 0.91 | 0.56 | 0.50 | 0.73 | 1.07 |
| Control | 20 | Weight | 17.37 | 6.99 | 12.98 | 16.91 | 20.58 |
|  |  | Length | 1.15 | 0.43 | 0.83 | 1.10 | 1.40 |
|  |  | Head Circumference | 0.75 | 0.47 | 0.47 | 0.73 | 1.00 |

P value for weight: 0.15; p value for length 0.09; p value for head circumference 0.57.
