# Supplementary material for: LioNeo project: a randomised double-blind clinical trial for nutrition of very-low-birth-weight infants
Source: Br J Nutr. 2022 Feb 11;128(12):2490–7. doi: 10.1017/S0007114521005110 (PMC9723485; doi:10.1017/S0007114521005110)
Supplement: Supplementary file 1 [file S0007114521005110sup.zip › S0007114521005110sup002.docx]

**PROTOCOL FOR ENTERAL NUTRITION OF PRETERM NEWBORNS**

**HC CRIANÇA – HC-FMRP-USP**

**Prof. Dr. José Simon Camelo Junior**

**Introduction**

Feeding preterm newborns (PTN, <37 weeks of gestational age) is a nutritional urgency and depends upon, essentially, their clinical condition. Technological advances have permitted the survival of even smaller newborns, in more serious and severe conditions, their behaviour is not homogeneous, and their nutrition becomes more and more complex, due to their lower weight, gestational age and immaturity.

Ideally, the physiological way, i.e., digestive system, should be used, but it is not always possible from the beginning, being necessary total or partial parenteral nutrition.

The milk to be used is human milk, preferably mother´s own milk (MOM). Initially colostrum MOM, but there are some limitations that will be discussed ahead, especially maternal *status* about cytomegalovirus (CMV). Since there is no MOM available, or insufficient, it must be used pasteurized banked human milk (BHM).

PTN can be classified in:

1. Low birth weight (LBW): BW<2500g;
2. Very low birth weight (VLBW): BW<1500g;
3. Extremely low birth weight (ELBW): BW<1000g.

**Objectives / Goals for Nutrition of PTN**

The Committee on Nutrition of the American Academy of Pediatrics (1985), defined intrautero growth velocity as the main goal for preterm newborn’s nutrition, according to adequate post conceptional age (AAP, Committee on Nutrition, 1985). Therefore, it is necessary plotting anthropometrical parameters of the neonate with appropriate intrauterine growth charts. Nowadays, we are using charts from the International Consortium Intergrowth 21st (<https://intergrowth21.tghn.org/>). New Fenton (Fenton et al, 2013) can be used for post natal growth, as well.

It is important to stress that post natal undernutrition of the preterm newborn can cause decrease in number of neurons, inducing a long term negative impact: on behaviour, cognitive aspects, memory ability and worsening preexisting or concurrent lesions in the Central Nervous System, such as intra and periventricular haemorrhage and leukomalacia.

On the other hand, DOHaD – developmental origins of health and disease – suggests that growing with an excessive speed may facilitate deleterious nutritional programming, allowing later development of degenerative diseases by epigenetic mechanisms, mainly metabolic syndrome (obesity, type 2 diabetes, dyslipidemia, systemic arterial hypertension).

Therefore, the main goals of the preterm newborn shall consider growing within an adequate velocity, mirroring, as much as possible, what would happen in utero, having consciousness of future impact, with good neurological development, minimizing previous damages and respecting the limitations of the prematurity.

**Initial Nutrition of LBWI, VLBWI, ELBWI**

- Low, Very low or Extremely Low Birth weight infants (LBWI, VLBWI, ELBWI) admitted to Neonatal Intensive Care Unit (NICU) or Intermediate Care Unit for Neonates (ICUN) must preferably receive colostrum from his/her own mother (MOM) and, if not possible, pasteurized colostrum from Human Milk Bank (HMB), as soon as possible (first or second day of life). Subsequentially, the newborn would ideally receive their MOM and/or mature pasteurized donated human milk. If there is an absolute formal contraindication to breastfeeding, infant formula can be used for preterm.
- Neonate must have a haemodynamically stable condition: acceptable blood pressure or with signs of stability, such as good peripheral perfusion (< 3 seconds), palpable peripheral pulse and normal oxygen saturation (90-95%).
- Newborns with asphyxia, are more likely to develop necrotising enterocolitis, due to central blood flow deviation/centralization, in order to protect coronary arteries, adrenal glands and Central Nervous System; in that cases the beginning of the nutrition can be postponed, by medical criteria, normally between second and fifth days of life, depending on the severity and complications of the asphyxia.
- If the neonate is between 32 and 34 weeks of post conceptional age has no respiratory distress, may receive colostrum at an Initial volume of 20 ml/kg using a cup and/or breastfeeding. It is important to previously test suctioning-swallowing-respiration coordination.
- If the neonate is <32 weeks of post conceptional age, and/or show respiratory distress at birth with any gestational age, a nasogastric or orogastric tube must be positioned, notwithstanding the ventilatory support and the colostrum must be administered by the gastrointestinal (GI) tube at an initial volume of 20 ml/kg/day.
- Abdominal circumference must be measured daily. If abdominal distention is suspected, with measurement >10% of the baseline, changing abdominal aspect, with reddish or purple colour, visible peristalsis and pain, associated or not to vomiting, abnormal stools (with blood, mucus), the nutrition can be suspended by medical criteria. Residues shall be tested only in abnormal situations, not routinely.
- The milk will be administered by bolus through syringes, by gavage or if necessary using infusion pumps (in the presence of vomiting, abdominal distention related to speed of milk infusion).
- Interval proposed to newborns>1000g: every 3 hours; every 2 hours can be considered by medical criteria, depending upon the tolerance of the diet. For newborns <1000g, see Table 2.

**Velocity of Milk Progression (Banked Human Milk – BHM – for <28 weeks GA or Raw MOM for >28 weeks GA)**

Table for diet progression proposed for newborns>1000g, adapted from *Emory University School of Medicine, Atlanta, GA, USA* (Ravi Patel, presentation in Brazilian Congress of Perinatology, Natal, RN, Brazil, 2018):

**Table 1:**

| **Day of Enteral Nutrition** | **Volume:**  **mL/kg/day** | **Concentration** | **Comentários** |
| --- | --- | --- | --- |
| 1 | 20 | BHM/MOM | Initially consider trophic feeds, if the evolution is adequate it can progress, if not, maintain volume |
| 2 | 50 | BHM/MOM | Attention to abdomen, vomiting, abdominal circumference daily |
| 3 | 80 | BHM/MOM | Attention to abdomen, vomiting, abdominal circumference daily |
| 4 | 80 | BHM/MOM | Consider the beginning of additive (human milk lyophilisate – LioNeo, or FM85® - Nestlé®) |
| 5 | 100 | BHM/MOM + additive | LioNeo, or FM85® - Nestlé® – Observe abdomen and vomiting carefully |
| 6 | 120 | BHM/MOM + additive | LioNeo, or FM85® - Nestlé® – Observe abdomen and vomiting carefully |
| 7 | 140 | BHM/MOM + additive | LioNeo, or FM85® - Nestlé® – Observe abdomen and vomiting carefully |
| 8 | 160 | BHM/MOM + additive | LioNeo, or FM85® - Nestlé® – Observe abdomen and vomiting carefully |

We present thereafter, Table 2 for progression of the diet for newborns<1000g at birth, based on *The Hospital for Sick Kids Protocol, Toronto, Canada* and associated hospitals (O’Connor et al, 2018).

**Table 2:**

| **Weight at birth** | **500-599g** | **600-699g** | **700-799g** | **800-899g** | **900-1000g** |
| --- | --- | --- | --- | --- | --- |
| Trophic feeds for induction | 1mL q4h/3days  1ml q3h/3days | 1mL q4h/3days  1ml q3h/3days | 1mL q4h/2days  1ml  q3h/2days | 1mL  q4h/2days  1ml  q3h/day | 1mL  q3h/2days |
| Velocity of increase of the diet | 1mL/2days | 1mL/day | 1mL/day | 1mL/day | 2mL/day |
| Aos 100mL/kg/dia | Start additive | Start additive | Start additive | Start additive | LioNeo or  FM85® |
| Aos 120mL/kg/dia | Simplify TPN | Simplify TPN | Simplify TPN | Simplify TPN | LioNeo or  FM85® |
| Aos 140mL/kg/dia | Suspend TPN | Suspend TPN | Suspend TPN | Suspend TPN | LioNeo or  FM85® |
| Aos 160mL/kg/dia | Maximum volume | Maximum volume | Maximum volume | Maximum volume | LioNeo or  FM85® |

TPN: total parenteral nutrition.

- If the infant gain weight higher than 1000g, use criteria from Table 1.
- GI enteral tubes must be positioned into the Treitz angle only if there is no success with gastric position, with vomiting of difficult control or severe gastro esophageal reflux. It is important to warn, that the enteral tube overpass the gastric barrier, increasing risk of bacterial translocation and/or late onset sepsis. Diagnosis of severe gastro esophageal reflux uncontrolled by medicines can indicate enteral GI tube. The correct enteral position must be when the GI tube reaches Treitz angle, after the fourth portion of duodenum; the nurse must measure crown-heel distance, put a mark in the GI tube, progress quickly to the stomach and slower up to jejunum entrance; confirm position by X-ray; in this position, milk must be administered slowly by infusion pump, due to osmolality.

**Transition of the Diet from GI Tube to Oral Feeding**

The transition for oral feeds must be done between 32 and 34 weeks of post conceptional age, when the infant has no ventilatory support, full feeds (160ml/kg/d), and assessment from a speech therapist of the suction-swallowing-respiration process. If it is adequate, infant can receive BHM/MOM by cup and breastfeed under supervision.

**Special Situations**

- If the neonate shows abdominal distention ≥10% of the baseline measurement, residues can be checked. If the aspect is clear or milky, volume can be maintained or step back, with a larger interval (q3h for q4h for example). If the aspect is abnormal, green, bloody, dark or fecaloid, diet must be suspended and GI tube must be kept opened for drainage. If abdominal aspect is reddish, tense, painful, followed or not by vomiting, abnormal residues and/or abnormal evacuations, diet must be suspended, rule out necrotising enterocolitis and put the newborn back on TPN.
- Due to potential risk of vertical transmission of CMV, raw milk (MOM) must only be utilized in newborns older than 28 weeks of post conceptional age.
- Umbilical catheters, CPAP and mechanical ventilation are not contraindications for feeding newborn infants by GI tubes (gastric or enteral). CPAP can facilitate abdominal distention by excess of air into GI system.
- Diet has only to be suspended in the situations previewed in the anterior paragraph. Venous catheters should not be kept in the hepatic territory otherwise the preterm should not receive enteral feeding.
- Infants presenting repeated episodes of apnea with bradicardia must have the continuity of the diet assessed, in order to not increase risk of bad perfusion of the bowel and consequently necrotising enterocolitis.
- Newborns under vasoactive drugs can receive diet by GI tube only if there is haemodynamic stability.
- Even though is controversial, diet must be suspended during blood product transfusions, returning afterwards.
- Newborns with mild abdominal distention, not abnormal residues and vomiting may receive trophic feeds, with total volume between 10 a 20 ml/kg/dia, during a defined period, generally from one week to 10 days, not increasing daily, with intention to stimulate GI mucosae trophism and motility of the bowel as well.
- Once the newborn has stability and conditions, it is encouraged to be positioned with his/her mother on kangaroo initiative; thus, has to be evaluated the suction-swallowing-respiration process, and since is considered adequate and safe, between 32 to 34 weeks of post conceptional age, may be breastfed and, if necessary, complemented with BHM, added or not with additives. Preterm formulas will be used only in the unavailable MOM or BHM.

**Referências**

1. Patel RM. Is possible to prevent necrotizing enterocolitis? Conference on 24^th^ Brazilian Congress of Perinatology. Natal, RN, Brazil.
2. Fenton TR, Kim JH. A systematic review and metanalysis to revise the Fenton growth chart for preterm infants. *BMC Pediatrics* 2013, 13:59-71.
3. Bertino E, Coscia A, Mombrò M, Boni L, Rossetti G, Fabri C, et al. Postnatal weight increase and growth velocity of very low birthweight infants. *Arch Dis Child Fetal Neonatal Ed* 2006; 91:F349–F356.
4. O’Connor DL, Kiss A, Tomlinson C, Bando N, Bayliss A, Campbell DM, et al. Nutrient enrichment of human milk with human and bovine milk-based fortifiers for infants weighing <1250g: a randomized clinical trial. *Am J Clin Nutr* 2018;9:1–9.
5. Rigo J, Hascoët J-M, Billeaud C, Picaud J-C, Mosca F, Rubio A, et al. Growth and nutritional biomarkers of preterm infants fed a new powdered human milk fortifier: a randomized clinical trial*. Journal of Pediatric Gastroenterology and Nutrition* 2017;65: e83–e93.
6. Villar J, Papageorghiou AT, Pang R, Ohuma EO, Ismail LC, Barros FC, et al. The likeness of fetal growth and newborn size across non-isolated populations in INTERGROWTH 21^st^ Project: The Fetal growth longitudinal study and newborn cross-sectional study. Lancet Diabetes Endocrinology 2014; 2(10):781-92.
7. Papageorghiou AT, Ohuma EO, Altman DG, Todros T, Cheikh Ismail L, Lambert A, et al. [International standards for fetal growth based on serial ultrasound measurements: the Fetal Growth Longitudinal Study of the INTERGROWTH-21st Project.](https://www.ncbi.nlm.nih.gov/pubmed/25209488) Lancet. 2014; 384(9946):869-79. Erratum in: Lancet. 2014; 384(9950):1264.
8. Villar J, Cheikh Ismail L, Victora CG, Ohuma EO, Bertino E, Altman DG, et al. [International standards for newborn weight, length, and head circumference by gestational age and sex: the Newborn Cross-Sectional Study of the INTERGROWTH-21st Project.](https://www.ncbi.nlm.nih.gov/pubmed/25209487) Lancet. 2014; 384(9946):857-68.
