# Supplementary material for: LioNeo project: a randomised double-blind clinical trial for nutrition of very-low-birth-weight infants
Source: Br J Nutr. 2022 Feb 11;128(12):2490–7. doi: 10.1017/S0007114521005110 (PMC9723485; doi:10.1017/S0007114521005110)
Supplement: Supplementary file 1 [file S0007114521005110sup.zip › S0007114521005110sup001.docx]

**Supplement 1**

**DONATED HUMAN MILK – RAW**

Selection and classification processes:

- Check for dirt

- Off-flavor

- Packaging conditions

- Color

- Dornic acidity

- Crematocrit

**50 ml HUMAN MILK RAW**

(Obs.: They will be frozen in an inert and sterile glass vial for at least 24hrs in a freezer at -30°C and then placed in the Lyophilizer L108®, LioTop to start the lyophilization cycle for 72hrs.)

**RECONSTITUTION WITH 75 ml HUMAN MILK RAW**

giving rise to the

**CONCENTRATE WITH HUMAN MILK LYOPHILISATE**

(Obs.: After being removed from the lyophilizer, the cold chain is maintained until they are reconstituted with raw human milk.)

**PASTEURIZATION**

(Obs.: the concentrates are placed in a water bath for 30 minutes at 62.5°C.)

**COOLING**

(Obs.: The concentrate is cooled in an ice bath until reaching 5°C and then kept in a cold chain until going to microbiological quality control.)

**MICROBIOLOGICAL QUALITY CONTROL**

**(bright green broth 5% w/v)**

**POSITIVE NEGATIVE**

**CONFIRMATORY PROOF OK - READY FOR CONSUMPTION**

**(bright green broth 4% w/v)**

**NEGATIVE**

Obs.: They will be sent frozen

to the dair of the Children´s Hospital, where they will be storaged.

**DISQUALIFIED FOR CONSUMPTION – DISCARD!**
